# The Dual Function of Reactive Oxygen/Nitrogen Species in Bioenergetics and Cell Death: The Role of ATP Synthase

**DOI:** 10.1155/2016/3869610

**Published:** 2016-03-10

**Authors:** Nina Kaludercic, Valentina Giorgio

**Affiliations:** Neuroscience Institute, National Research Council of Italy (CNR), Via Ugo Bassi 58/B, 35131 Padova, Italy

## Abstract

Reactive oxygen species (ROS) and reactive nitrogen species (RNS) targeting mitochondria are major causative factors in disease pathogenesis. The mitochondrial permeability transition pore (PTP) is a mega-channel modulated by calcium and ROS/RNS modifications and it has been described to play a crucial role in many pathophysiological events since prolonged channel opening causes cell death. The recent identification that dimers of ATP synthase form the PTP and the fact that posttranslational modifications caused by ROS/RNS also affect cellular bioenergetics through the modulation of ATP synthase catalysis reveal a dual function of these modifications in the cells. Here, we describe mitochondria as a major site of production and as a target of ROS/RNS and discuss the pathophysiological conditions in which oxidative and nitrosative modifications modulate the catalytic and pore-forming activities of ATP synthase.

## 1. Introduction

Reactive oxygen species (ROS) and reactive nitrogen species (RNS) play important physiological functions but can also cause extensive cellular damage, in a balance that is determined by their relative rates of formation and removal. Usually, these species are removed rapidly before they cause cell dysfunction and death. Oxidative/nitrosative stress generated by an imbalance between formation of ROS/RNS and antioxidant defense capacity can affect major cellular components, including lipids, proteins, carbohydrates, and DNA. Mitochondria are recognized as a critical site in the cell for the formation of ROS/RNS and as their target.

Mitochondrial processes are highly compartmentalized because of the existence of two limiting membranes allowing the selective localization of proteins, nucleotides, and coenzymes in the intermembrane and matrix spaces. The outer mitochondrial membrane (OMM) is the interface between mitochondria and the cell components and its permeabilization is essential to allow the release of mitochondrial proteins involved in apoptosis such as cytochrome c [1]. The inner mitochondrial membrane (IMM), whose permeability to solutes is controlled by highly specific transporters and tightly regulated channels, is the site of coupling between substrate oxidation and ATP synthesis in the process of oxidative phosphorylation. Mitochondria operate a sequence of energy conversion processes through which the exergonic flow of electrons along the respiratory complexes supports the endergonic pumping of protons from the matrix to the intermembrane space. The resulting proton motive force drives the rotation of the F_O_ sector of ATP synthase leading to the synthesis of ATP in the F_1_ sector, but the electron flow through the respiratory chain also generates ROS/RNS. In addition, the mitochondrial permeability transition pore (PTP), a large-conductance channel, is also located at the level of the IMM and prolonged opening of this channel leads to mitochondrial swelling, rupture of the OMM, and cell death [2]. PTP opening is dependent on the presence of matrix calcium, but the threshold calcium load which is required is modulated by inducers of the pore such as oxidants [3]. Although the existence of the PTP was established as early as the 1970s [4–6], its molecular nature has been the subject of controversy as many potential components were ruled out by the use of targeted gene deletion in mice [2]. The only candidate remaining is cyclophilin D (CyPD), which was found to act not as a structural component of the pore but as a modulator whose binding to the PTP decreases the threshold calcium concentration necessary to induce permeability transition [7–10]. CyPD was shown to interact with the lateral stalk of the ATP synthase in mammals [11], a finding which was the basis for the characterization of the molecular structure of the PTP as formed by ATP synthase itself [12–16]. Genetic ablation of the* Ppif* gene (which encodes for CyPD) in the mouse or its displacement from the PTP by the treatment with cyclosporin A (CsA), a known inhibitor of the PTP, has been also used to demonstrate the important role of PTP in the pathophysiological mechanism of several diseases such as neurodegenerative diseases, muscular dystrophies, ischemia/reperfusion (I/R), and diabetes [2, 17, 18]. Besides the PTP, mitochondrial function and bioenergetics (including the modulation of the catalytic activity of ATP synthase) are also affected in most of these pathophysiological conditions and ROS/RNS are presumably involved as causative factors.

While numerous mechanisms of oxidant-induced injury have been identified, the impact of oxidants on the mitochondrial proteome has been investigated only recently. Oxidative or nitrosative stress may not only alter levels of mitochondrial proteins, but also induce posttranslational modifications of proteins. These modifications involve reversible changes at the level of cysteine, tyrosine, methionine, histidine, and tryptophan residues and irreversible protein carbonylation [19]. Thiol groups can be S-nitrosylated by nitric oxide (NO) or reversibly oxidized by ROS to form disulfide bonds or sulfenic acid; the latter can be further oxidized to sulfinic and sulfonic acids [20]. Sulfenic acid can also interact with glutathione to become glutathionylated. Tyrosine residues instead are target for peroxynitrite (ONOO^−^) which leads to irreversible formation of 3-nitrotyrosine. All these modifications lead to changes in protein structure and/or activity, thereby affecting their roles in cell function. In this review we discuss the role of mitochondria as a source and target of ROS/RNS during the switch between cell death and survival. We will focus on the effects of these reactive species on critical ATP synthase amino acid residues as part of the mechanism affecting ATP synthesis and/or permeability transition.

## 2. Mitochondria Are Sources of ROS/RNS

A number of sites responsible for ROS/RNS formation within the cell have been identified and shown to play a role in different pathologies. NADPH oxidases, xanthine oxidase, uncoupled nitric oxide synthase (NOS), and mitochondria are all relevant sources of ROS that in certain pathological conditions contribute to oxidative damage of cells and tissues. Indeed, these processes have been identified as disease-relevant enzymatic sources of ROS and their inhibition has been shown to afford protection in a number of diseases both in experimental models and in patients [21, 22].

### 2.1. Mitochondrial Sources of ROS

It is well accepted that mitochondria are a major source of ROS in the cell. These organelles contain several enzymes that catalyze ROS formation either as the obligatory product or as the result of an occasional, possibly undesired, reaction [23]. The best example of “accidental” ROS formation is represented by the mitochondrial respiratory chain. A small fraction of the electrons (about 0.1%) flowing through the respiratory chain is diverted causing the partial reduction of O_2_ into superoxide [24]. This process occurs at the level of the first three complexes where flavins or quinones are able to act as single electron donors. The electron detour at these sites is favored when flow is hampered downstream as a result of either protein alterations in respiratory complexes or their inhibition. Other mitochondrial enzymes, such as flavin containing glycerol-3-phosphate-, proline-, and dihydroorotate-dehydrogenase, and the electron transferring flavoprotein/ETF:Q oxidoreductase system of fatty acid *β*-oxidation have also been described as potential ROS producers [25]. Nevertheless, all these enzymes and respiratory complexes normally catalyze reactions required for energy metabolism, cell function, and viability maintenance, making it difficult to envision their inhibition as a potential therapeutic tool.

Mitochondria also contain other enzymes that may generate hydrogen peroxide (H_2_O_2_) as a direct and obligatory product. One such example is p66^Shc^, cytosolic adaptor protein that upon stress translocates to mitochondria where it catalyzes electron transfer from cytochrome c to oxygen, a process that can result in the formation of ROS [37]. Genetic deletion of p66^Shc^ protects against I/R injury in mice [38, 39], obesity [40], and diabetic complications such as cardiomyopathy [41], nephropathy [42], delayed wound healing [43], pancreatic cell death [44], and endothelial dysfunction [45]. On the other hand, the observation that mice lacking p66^Shc^ actually live shorter when exposed to natural conditions (low temperatures and food competition) [46] may suggest that p66^Shc^ dependent ROS also serve a physiological role. NADPH oxidase 4 (Nox4), an enzyme belonging to the NADPH oxidase family, is another ROS generating enzyme that has been reported to localize in the plasma membrane and apparently also in the mitochondria, focal adhesions, nucleus, and endoplasmic reticulum [47]. This enzyme associates with p22^phox^ for its activation; it is constitutively active, and, unlike other members of the Nox family, generates H_2_O_2_ rather than superoxide [47, 48]. However, the relevance of Nox4 for mitochondrial ROS generation remains controversial. A recent study has shown that Nox4 can (in principle) interact with complex I subunits, but under physiological conditions Nox4 protein or ROS formation could not be detected in kidney or heart mitochondria [49]. Other studies have shown that cardiac-specific targeting of Nox4 can be both protective and harmful in different models of cardiac pressure overload [50, 51]. Moreover, while certain studies reported Nox4 to be deleterious, contributing to mitochondrial dysfunction and several pathologies such as ischemic stroke [52], diabetic cardiomyopathy [53], vascular inflammation, and remodeling [54], others concluded that Nox4 might be vascular-protective rather than vascular-damaging [55]. These apparently contradictory findings reflect the need for further investigation in order to address the pathophysiological role and regulation of Nox4. Another major source of H_2_O_2_ in the mitochondria is monoamine oxidase (MAO). Activation of this enzyme, localized at the level of the OMM, leads to H_2_O_2_ formation and has been shown to contribute to neuronal disorders, such as Parkinson's (PD) or Alzheimer's disease (AD), most likely due to formation of ROS responsible for oxidative damage to neurons [56]. In recent years, the contribution of MAO to oxidative stress that ultimately leads to mitochondrial dysfunction and cell damage has been demonstrated also in the cardiovascular field where MAO inhibition has been shown to be protective in I/R injury, pressure overload, vascular damage, and diabetes [23, 57–62].

### 2.2. Mitochondrial Sources of RNS

In addition to ROS, cells are also capable of generating RNS. NOS exist in three isoforms (endothelial (eNOS), neuronal (nNOS), and inducible (iNOS)) which catalyze the conversion of L-arginine into citrulline and NO. This reaction also requires flavin adenine dinucleotide, flavin mononucleotide, tetrahydrobiopterin (BH4), heme, and calmodulin. These cofactors are important (e.g., when BH4 levels are limited) because NOS may become uncoupled and generate superoxide instead of NO [63, 64]. Another possibility is that, in conditions of high oxidative stress, NO and superoxide interact to generate ONOO^−^, a very reactive species capable of nitrating tyrosine residues, thus amplifying oxidative damage.

It is generally accepted that NO has protective effects on mitochondria although it remains unclear whether mitochondria actually possess a mitochondrial NOS [65–69]. Nevertheless, in addition to being generated by NOS, NO can be formed from dietary inorganic nitrite (NO_2_^−^) and nitrate (NO_3_^−^) [70]. Several in vitro studies have demonstrated that mitochondria are able to metabolize NO_2_^−^ into NO, since cytochrome c and respiratory chain complexes III and IV possess nitrite reductase activities that can be stimulated under hypoxic or acidic conditions [71–73]. Either way, the bioactivity of newly synthesized NO is rapidly terminated by its oxidation into NO_2_^−^ and NO_3_^−^, thus completing the NO cycle. The protective effects exerted by NO on mitochondria are due to S-nitrosylation of critical cysteine residues. One such example is represented by the mechanism through which MitoSNO, a mitochondria selective S-nitrosylating agent, affords protection in cardiac I/R injury in vivo [74]. Administration of MitoSNO within the first minutes of reperfusion temporarily inhibits complex I and thus blocks the reverse electron flow that leads to generation of ROS, oxidative damage, and tissue necrosis [74, 75]. The inhibition of complex I is due to S-nitrosylation of a critical cysteine residue (Cys39) within the ND3 subunit, underlining the importance of (i) the reversibility of cysteine modification and (ii) mitochondria as both source and target of ROS/RNS. This latter notion is further supported by the observations that mice lacking Mn superoxide dismutase (SOD), enzyme responsible for conversion of superoxide into H_2_O_2_, develop ROS toxicity and dilated cardiomyopathy [76] and that targeting catalase expression in mitochondria affords beneficial effects in a number of pathologies [77–80].

## 3. The Role of ATP Synthase in Mitochondrial ROS Formation

Recent evidence suggests that ATP synthase activity can modulate ROS formation in mitochondria. Although not a direct source of ROS, ATP synthase is able to regulate energy metabolism and modulate pathways leading to ROS formation, cell death, and survival. A recent study by Ni et al. [81] very elegantly showed that *α* subunit is a target for calpain-1 in diabetic hearts, leading to its proteolytic degradation, reduction in ATP synthase activity, increase in mitochondrial superoxide formation, and diabetic cardiomyopathy in mice. Indeed, genetic inhibition of calpain-1 or upregulation of *α* subunit increased ATP synthase activity and reduced mitochondrial ROS generation and all the downstream changes occurring in diabetic hearts. It is likely that, besides insufficient ATP production that can directly contribute to myocardial dysfunction, disruption in ATP synthase activity leads to the accumulation of the electrons in the upstream complexes of the respiratory chain, promoting superoxide generation through complexes I and III [81].

Whereas inhibition of ATP synthase in normal cells leads to mitochondrial oxidative stress and cell death [81–83], most cancer cells are highly glycolytic and have adopted several molecular strategies to reduce oxidative phosphorylation including ATP synthase activity inhibition [84, 85], thus modulating the level of ROS in mitochondria. This does not only occur through downregulation of ATP synthase, but also through overexpression of its inhibitor protein (IF_1_) [86]. IF_1_ is the endogenous inhibitor of ATP synthase that reversibly binds to the enzyme. Its binding is promoted by low matrix pH and membrane potential, thereby limiting ATP hydrolysis and energy dissipation [87, 88]. In fact, its role has been extensively studied in myocardial ischemia and ischemic preconditioning (IPC), since IF_1_ seems to mediate ATP synthase inhibition and thus sparing of ATP during myocardial preconditioning [89–92]. Moreover, IF_1_ seems to regulate the oligomeric state of ATP synthase, increasing the density of cristae and formation of ATP synthase dimers [93–96]. Mitochondria from different types of carcinoma show a remarkable increase in IF_1_ expression [86] that has been linked to the inhibition of ATP synthase, glycolytic switch in energy metabolism, and production of ROS [97–99]. A critical role of the phosphorylation status of IF_1_ has been recently suggested in the modulation of ATP synthesis and respiration in tumors and heart [100]. ROS generated when ATP synthesis is inhibited are used as nuclear signals to initiate transcription of genes necessary to support tumor development [86, 97]. Indeed, IF_1_ triggers ROS-induced activation of transcription factors (such as NFkB and HIF1*α*) in cancer cells causing enhanced proliferation, invasion, and survival [97]. These findings highlight how cancer cells, as opposed to normal cells, rely on a subtle mechanism of redox equilibrium: on the one hand, ROS enhance proliferation and favor genomic instability by damaging DNA, while on the other hand, excess ROS is harmful as it could promote PTP opening and cell death [101]. Moreover, it is tempting to speculate that PTP might be differently modulated by IF_1_ and CyPD in cancer cells, as observed for CyPD in osteosarcoma and prostatic cell models [102]. This suggests that this different PTP modulation might render cancer cells more resistant to cell death. Nevertheless, the ROS signaling pathways triggered by IF_1_ overexpression in many other cancer types still remain to be elucidated.

## 4. Mitochondrial ROS/RNS Induce Posttranslational Modification of ATP Synthase Residues

**Figure 1:**
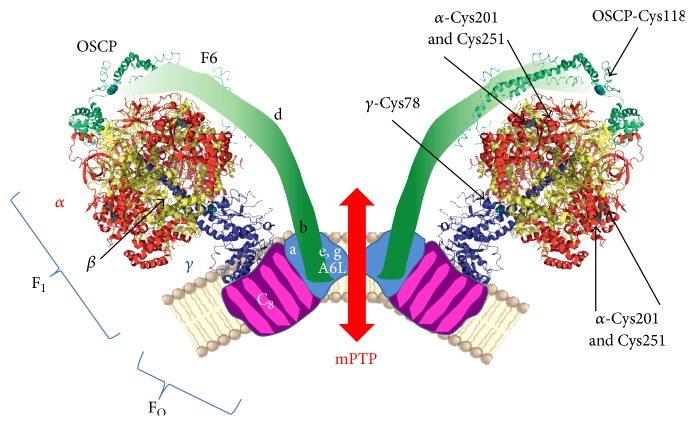
Schematic representation of ATP synthase and PTP. Dimers of ATP synthase that form the PTP are shown from a lateral view. F_1_ catalytic part of ATP synthase is from bovine crystal structure (PDB 2WSS, modified by PyMOL 1.3 software) and is composed of *α*, *β*, and *γ* subunits in red, yellow, and blue, respectively, as indicated in the left part of the dimer. F_O_ and lateral stalk subunits are also indicated in the left part in pink, light-blue, and green regions. On the right part of the dimer arrows indicate the critical cysteine residues modified by ROS/RNS in pathophysiological conditions. Cysteines are highlighted in cyan.

ROS/RNS induced posttranslational modifications are known to modulate the catalytic activity of ATP synthase in several pathophysiological conditions. Matrix calcium levels and oxidative/nitrosative stress play a crucial role also in the modulation of PTP. Recent finding that ATP synthase dimers form the PTP [12] makes the identification of the sites involved in the two different functions of ATP synthase more feasible (Figure 1). The most frequently modified sites of ATP synthase possibly involved in mitochondrial pathophysiology are listed in Table 1.

### 4.1. Matrix Residues

Critical thiols facing the matrix are involved in the modulation of ATP synthase catalysis. H_2_O_2_ inactivates F_1_-ATPase activity from bovine heart through formation of iron-protein adducts [103]. ROS- and RNS-mediated modifications responsible for ATPase inactivation are mostly localized at the level of cysteine residues in the *α* and *γ* subunits [104]. A special feature of higher plant CF_1_-ATPase is a regulatory domain in the *γ* subunit consisting of three methionines and a cysteine that were identified by mass spectrometry to be oxidized by ROS. Reduction of the disulfide bonds of *γ* subunit elevates ATP hydrolysis and synthesis [105].

Although in mammalian cells the chloroplast redox-sensitive *γ* subunit region is not present, mitochondrial ATP synthase F_1_ domain might be a major site for oxidative/nitrosative posttranslational modifications. Garcia et al. [27] have reported that *α* subunit is S-glutathionylated under oxidative stress in rat brain or liver mitochondria leading to decreased ATPase activity. The same inhibitory effect due to *α* subunit S-glutathionylation was observed in transgenic mouse hearts overexpressing iNOS [28]. Sun et al. [29], on the other hand, reported inhibition of ATPase activity which is protective in preconditioned mouse hearts during myocardial ischemia due to *α* subunit S-nitrosylation and an additive cardioprotective effect of this posttranslational modification which causes desensitization of the PTP [30]. An age-associated decline of ATPase activity due to carbonylation of *α* subunit was also observed in mouse skeletal muscle [31]. An indirect effect of ROS on *α* subunit was also found following H_2_O_2_ treatment of mitochondria that caused tyrosine-phosphorylation of *α* (but not *β*) chain [32]. This is likely due to the inactivation of phosphatases mediated by H_2_O_2_ [106].

**Figure 2:**
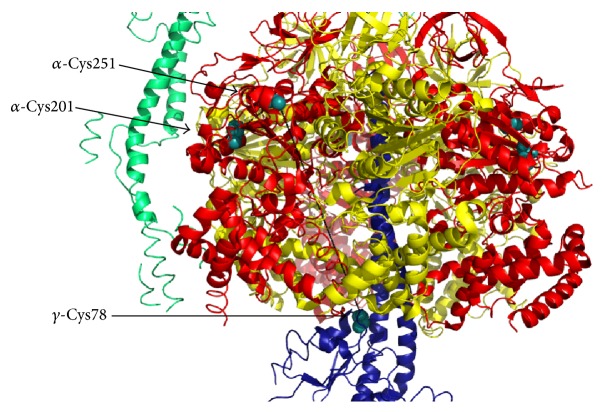
Lateral view of a section of the catalytic core of ATP synthase (PDB 2WSS, modified by PyMOL 1.3 software) composed of *α*, *β*, and *γ* subunits in red, yellow, and blue, respectively. Critical cysteine residues subjected to posttranslational modifications are highlighted in cyan. Distances between *α*-Cys251 and *α*-Cys201 or *α*-Cys251 and *γ*-Cys78 are indicated by black dashed lines and are 12 Å or 61.5 Å, respectively.

**Figure 3:**
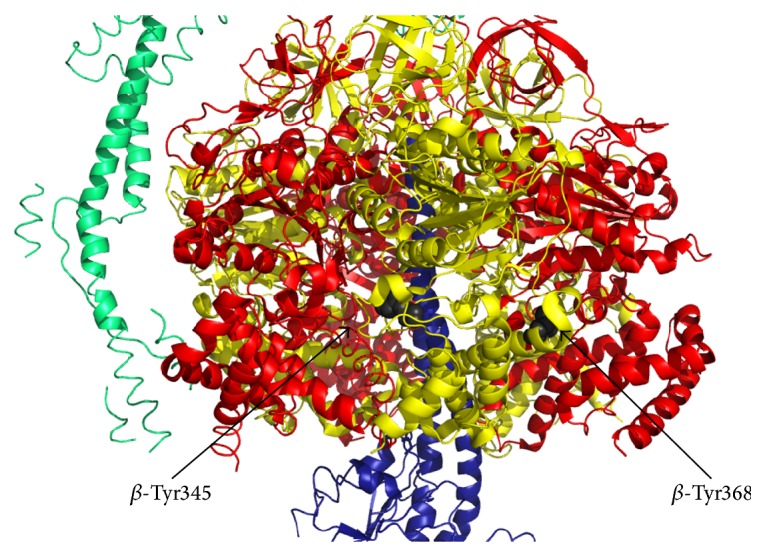
Lateral view of a section of the catalytic core of ATP synthase (PDB 2WSS, modified by PyMOL 1.3 software) composed of *α*, *β*, and *γ* subunits in red, yellow, and blue, respectively. Critical *β*-Tyr345 and *β*-Tyr368 residues that might be modified by RNS are highlighted in gray.

Two disulfide bonds were identified between cysteines of ATP synthase *α* and *γ* subunits and between those of *α* subunits in heart failure [26] and both S-glutathionylation and S-nitrosylation of *α*-cysteine 294 (corresponding to bovine Cys251, Figures 1 and 2) can prevent the formation of these disulfide bridges. Other selective targets of ROS in the matrix are tryptophan residues of d subunit of ATP synthase in mammals [36] or tryptophan 503 of *α* subunit in* Podospora anserina* [107]. In bovine heart mitochondria treated with ONOO^−^, 3-nitrotyrosine modified residues were identified at the level of *β* and d subunits of ATP synthase [108, 109]. Although it is not yet known which of the tyrosine residues present in this subunit were modified, one might speculate that Tyr345 and Tyr368 in the *β* subunit are involved, since they have been suggested to be a major target for nitrosative stress in rat liver under in vivo conditions [35] (Figure 3). Carbonylation at the level of *β* subunit was observed in* Escherichia coli* after treatment with H_2_O_2_ [110]. Whether sites targeted by ROS/RNS that modulate ATP synthase are also involved in PTP formation/modulation has not been clarified yet.

The first indication of the presence of ROS-sensitive modulatory thiols on the matrix-facing side of the PTP was revealed in isolated mitochondria through the use of chemical thiol-oxidants, such as diamide, arsenite anion (AsO), copper-*o*-phenanthroline, phenylarsine oxide (PhAsO), and *β*-hydroxybutyrate, or thiol-modifiers, such as* N*-ethylmaleimide (NEM) or monobromobimane (MBM) [111–114]. In these studies the authors proposed that the PTP can be modulated by oxidation-reduction effectors at two sites that can be distinguished experimentally. One site (which was called the “P site”) appears to be modulated through the oxidation-reduction state of pyridine nucleotides even when glutathione is fully reduced and can be blocked by NEM, but not by MBM. The other site (which was called the “S site”) coincides with the oxidation/reduction-sensitive dithiol(s) [111], and it can be activated by reaction with AsO or PhAsO. The S site can be blocked by both NEM and MBM. Irrespective of the precise mechanism by which glutathione and pyridine nucleotides affect the PTP, oxidative stress causes an increased probability of pore opening when the concentrations of reduced glutathione and pyridine nucleotides decrease.

Some of the above-mentioned oxidants and thiol-modifiers of the PTP sites are also known to be reagents effective on ATP synthase [115] and since the PTP is formed by ATP synthase dimers, an accurate analysis of the amino acid residues of ATP synthase might clarify the molecular localization for the S and P sensitive sites of the PTP. Moreover, the characterization of these residues could be useful to discriminate between the two effects of ROS/RNS on ATP synthase: the modulation of its catalytic activity and the induction of the PTP.

Upon treatment of beef heart mitochondria with radioactive NEM and dithiobis(nitrobenzoate), these radiolabeled compounds were incorporated into the oligomycin-sensitivity conferring protein (OSCP) subunit, which faces the matrix side, through its only cysteine (OSCP-Cys118 in Figure 1) without any detectable effect on the ATPase activity [34, 116]. Intriguingly, the OSCP subunit has been identified as the binding site for CyPD to ATP synthase [12], an interaction favored by phosphate [11]. In mouse liver mitochondria high phosphate (5 mM) protects PTP from the oxidative stress, something that is not observed in CyPD knockout (KO) mitochondria [8], suggesting that the binding of CyPD on OSCP, which is favored by phosphate, might affect PTP thiols availability. Taken together, these results suggest that the OSCP subunit in mammals might play a crucial modulatory role for PTP opening and ROS sensitivity. This hypothesis might explain the previous and never fully understood finding that overexpression of CyPD in HEK293 cells protects from oxidative stress and apoptosis [117].

### 4.2. Intermembrane Space/IMM Residues

Many possible sites of oxidation are present in the F_O_ sector and in the noncatalytic subunits of ATP synthase in the IMM and facing the intermembrane space (Figure 1). These conserved cysteines are involved in dimer/oligomer formation.

Preferential interactions in yeast dimers occur through subunits e [118, 119], g [120], 6 [121], and 4 [122] and also through subunit h and the yeast-specific subunit i [123], most of which harbor cysteine residues. Among the mammalian corresponding subunits e, g, a, b, and F6 (Figure 1) only b presents a conserved cysteine residue. However, in both yeast [121, 124] and mammals [95, 125, 126], the stabilizing contribution of the different subunits seems to be additive. In yeast mutants lacking e and/or g subunits, the PTP was desensitized to opening [14]. These data support the hypothesis that dimerization of ATP synthase is necessary for PTP formation. Moreover, the effect of ROS is conserved from human to yeast and* Drosophila* [127], since dimers extracted from blue native gels and tested for their channel activity generated currents in the presence of oxidants in all these species [12, 14, 15]. In yeast, both e and g subunits harbor a cysteine residue (eCys28 and gCys75) and form e/g interactions in the dimerization interface through GXXXG motifs [117]. They also participate in the putative oligomerization interface through e/e and g/g interactions [124], as established in a yeast gCys75Ser/Leu109Cys mutant, which formed eCys28-eCys28 and gLeu109Cys-gLeu109Cys cross-links [118, 120]. A similar arrangement of e and g subunits in the IMM of bovine heart has been hypothesized based on cross-linking experiments [128]. The treatment of yeast mitochondria lacking e and/or g subunits with copper(II) chloride (CuCl_2_), which mimics the effect of oxidants, stabilized preexisting dimers by formation of disulfide bridges between adjacent monomers [14]. This suggests the involvement of species-specific cysteine residues located in subunits other than e and g that might stabilize dimers, as it was observed in subunit 6 in yeast [124] or factor B [129] and subunit b in bovine heart, whose modification also affects enzyme function [130–132]. Subunit b spans the membrane without contacting the c_8_-ring in the enzyme monomer, suggesting that two subunits b are in close proximity in the dimer [133].

It still remains to be defined whether these or other ATP synthase residues might coincide with cysteines of the PTP facing the intermembrane space showed to be sensitive to membrane-impermeant photooxidants in isolated mitochondria [134]. Moreover, their involvement in the modulation of the PTP in mitochondrial pathophysiology in humans remains to be addressed.

## 5. PTP Modulators as Targets for ROS/RNS

S-nitrosylation of CyPD cysteine 203 seems to be important for the modulation of the PTP during I/R injury [135–137], since its modification inhibits PTP opening. Moreover, the substitution of this residue with a serine desensitizes PTP in MEF cells exposed to oxidative stress similarly to CyPD ablation [137]. Indeed, cysteine 203 has been identified as a major cysteine residue susceptible to oxidation in human cells, leading to conformational changes of CyPD and induction of PTP [138]. Another possible effect of ROS on the modulation of the PTP is the direct phosphorylation of CyPD by mitochondrial glycogen synthase kinase 3 (mGSK3) [102, 139]. This kinase has been involved in the regulation of PTP in pathological conditions, such as cardiac I/R injury or hypoxia [140–144], in tumor cells [102, 139], and in cells lacking mtDNA [145]. ROS-dependent activation of mGSK3 enhances cell death in neurons, probably following PTP induction [146, 147], and this suggests that mGSK3 coordinates diverse signaling pathways to connect PTP opening with stress and survival signals by modulating ROS and/or calcium threshold of PTP opening.

Another recently identified interactor of CyPD that might be involved in the modulatory effect of ROS on the PTP is the IMM protein spastic paraplegia 7 (SPG7), but the mechanisms of action through which it could sensitize the PTP to opening in the presence of oxidative stress need to be clarified [148].

## 6. The Dual Role of ATP Synthase in Cardiac Disease

The cardiac muscle heavily depends on mitochondrial bioenergetics and metabolism for its function. This becomes particularly evident during I/R, a scenario in which low levels of oxygen lead to the inhibition of electron flow through the respiratory chain and thus an impairment of energy conservation and oxidative metabolism. The resulting loss in proton motive force prevents ADP phosphorylation to ATP at the level of ATP synthase, which rather works in “reverse mode” coupling ATP hydrolysis to proton pumping. The net result is that mitochondria no longer produce ATP and become very powerful in hydrolyzing glycolytic ATP [149]. IMM permeabilization due to the opening of the PTP leads to depolarization and worsening of ATP depletion and precipitates cell death [150]. Indeed, the crucial role of the pore opening in mediating I/R induced damage was extensively corroborated by the protective effects of CsA [151] and lack of injury in CyPD KO mice [7, 9]. The opening of the PTP was thought for years to occur due to mitochondrial calcium overload, but this hypothesis was questioned recently by the observation that MCU KO mice are not protected from I/R injury [152]. Nevertheless, recent reports showed that cardiac-specific ablation of MCU affords protection from I/R injury [153, 154]. Considering that MCU KO MEFs are equally susceptible to oxidative damage as their wild type counterparts [152], it is tempting to speculate that PTP opening might be induced by oxidative stress independently of mitochondrial calcium overload, either by directly targeting critical cysteine residues of ATP synthase to induce conformational changes and dimer formation or through oxidative changes of CyPD. Other amino acid residues might also be involved in the oxidative modulation of the enzyme activity and/or conformation, such as tyrosine nitration in the *α* subunit occurring in cardiac I/R injury [155] or tryptophan oxidation described at the level of subunits d and a [36]. The functional consequences resulting from these modifications are yet to be elucidated.

It is well accepted that low levels of ROS are key signals mediating physiological or cardioprotective responses, whereas high levels of ROS contribute to oxidative stress and cell or tissue damage. This dual effect has been named mitohormesis [156, 157]. One such example of beneficial effects of ROS is represented by IPC. IPC consists of brief periods of ischemia followed by brief periods of reperfusion, which prepare the heart and render cardiomyocytes more resistant to sustained ischemic insults. Although IPC involves complex mechanisms that are not completely understood, it has been established that ROS/RNS mediate signal transduction in the early phase of IPC through posttranslational modifications of redox-sensitive proteins [158]. Again, mitochondrial proteins come into play as both the source and the target of these species. More specifically, IPC resulted in the S-nitrosylation of subunit *α* [30]. S-nitrosylation has been suggested to be protective, shielding the cysteine residues from oxidation and thus protecting the proteins from irreversible oxidation occurring at the start of reperfusion. It has also been reported that S-nitrosoglutathione (GSNO) treatment, also protective against I/R injury, leads to S-nitrosylation of the *α* subunit and this results in decreased ATP hydrolytic activity [29]. Thus, in addition to shielding the cysteine residues from irreversible oxidative damage, S-nitrosylation might also afford protection by decreasing ATP synthase activity and therefore reduce the consumption of ATP following myocardial ischemia. S-nitrosylation of ATP synthase subunit *α* occurs in both IPC and postconditioning and is dependent on NOS activity [30, 159]. Moreover, cysteine 19 of the subunit *ε* was also found to be S-nitrosylated following postconditioning but the functional significance of this finding remains to be elucidated [159].

Oxidation and S-nitrosylation have also been shown to occur at the level of ATP synthase in a canine model of dyssynchronous heart failure (DHF) [26]. Indeed, DHF was correlated with disulfide bond formation in the ATP synthase complex occurring between either cysteine 294 of the neighboring *α* subunits or cysteine 294 of the *α* subunit and cysteine 103 of the *γ* subunit. This cross-linking was negatively correlated with ATP hydrolytic activity. Furthermore, cysteine 294 of the ATP synthase *α* subunit was also S-glutathionylated in DHF, another modification that correlated with a reduction in ATP synthase activity [104]. Interestingly, during chronic resynchronization therapy (CRT, the only clinically effective therapy for DHF) the disulfide bond at cysteine 294 was found to be reversed and partially replaced by S-nitrosylation, resulting in the recovery of ATPase activity. Indeed, the same group has shown that cysteine 294 is critical for ATP synthase function in vitro and that it may play a major role in redox regulation of ATP production acting as a redox-sensor [160]. Nevertheless, the S-nitrosylation of the *α* subunit and improvement in ATPase activity observed in this study are in contrast with the previously mentioned results from Sun et al. [29], showing that S-nitrosylation of ATP synthase *α* subunit in IPC or after GSNO treatment leads to a reduction of its hydrolytic activity. One cannot exclude that different cysteine residues were modified in the two aforementioned studies, a fact that could explain this discrepancy. Another possible explanation is that cross-link reversal and recovery of ATPase activity observed in CRT occur at a higher level compared to the extent of S-nitrosylation, suggesting that the majority of cysteine residues are actually free in CRT hearts [26]. On the other hand, the distances between the two cysteines 294 of neighboring *α* subunits or between *α* cysteine 294 and *γ* cysteine 103 are more than 5 Å (corresponding to *α*-Cys251 and *γ*-Cys78, respectively, in Figures 1 and 2), making it unlikely that these disulfide bonds actually occur in the assembled complex. Rather, these bonds could form in the misfolded/aggregated enzyme, suggesting that the reversal of cysteine cross-links observed in CRT may promote the correct assembly of the complex, thus resulting in improved ATPase activity. Of note, CuCl_2_ treatment of isolated ATP synthase complex from rat mitochondria in vitro showed that disulfide bonds can form also between *α* and OSCP subunits as well as between the two *γ* subunits and *γ* and OSCP [26]. The effect of these disulfide crossbridges on ATP synthase activity was not addressed, and whether these modifications could also occur in vivo still needs to be assessed. Further efforts should also be put in addressing the effect of these oxidative posttranslational modifications on PTP formation.

## 7. Neurodegenerative Diseases

Mitochondria have a central role in aging-related neurodegenerative diseases. Oxidative stress generated by mitochondria has been inversely correlated with longevity in model organisms [161, 162] and defects in mitochondrial bioenergetics have been implicated in a number of neurodegenerative diseases [163].

In vitro, ATP synthase is susceptible to ROS [103, 164, 165] and to oxidative/nitrosative stress associated with disorders of the central nervous system [166, 167] and aging [35, 168]. The fact that ROS/RNS modifications can alter the mitochondrial oxidative phosphorylation efficiency may explain the mitochondrial involvement in neurological diseases.

Moreover, the involvement of PTP in neurodegenerative diseases has been demonstrated by the use of CsA both in vitro and in vivo and the ablation in mouse models of its target, CyPD [17, 18]. CyPD plays an important dual function on the modulation of ATP synthase; on the one hand, it sensitizes PTP to matrix calcium, while, on the other hand, it inhibits both ATP hydrolysis and synthesis by 30% [11]. In cellular mechanisms of neurodegenerative diseases, when mitochondria are hydrolyzing ATP, CyPD binding and inhibition of ATP synthase can be an advantage avoiding ATP consumption and cell death, or a disadvantage in cases in which PTP is sensitized to calcium and the displacement of CyPD would promote cell survival. This is only one of the complex relationships between ATP synthase and PTP in neurodegeneration, and in this paragraph we attempt to summarize possible mechanisms linking ATP synthase/PTP and ROS/RNS modifications.

### 7.1. Amyotrophic Lateral Sclerosis

Amyotrophic lateral sclerosis (ALS) is a progressive degeneration of motor neurons. In familial ALS, mutations have been found in Cu,Zn-SOD that are suggested to increase generation of ROS [169, 170], RNS, and nitrosylation [171]. Furthermore, Beal et al. [172] detected increased levels of nitrotyrosine staining in motor neurons of both sporadic ALS and familial ALS, suggesting that ONOO^−^ mediated oxidative damage may play a role in both forms of the disease. Of note, as already mentioned, tyrosine modifications at the level of the *β* subunit of ATP synthase have been described to be modulatory of the catalytic ATPase activity in the presence of ROS/RNS [108, 109], even if their direct involvement in ALS remains to be addressed.

In light of the susceptibility of the mitochondrial respiratory chain to nitrosative stress, it is not surprising that mitochondrial function is impaired in ALS [173]. Spinal cord mitochondria in ALS mouse model display decreased calcium retention capacity long before the onset of motor weakness and neuronal death [174], and this was corrected by ablation of the* Ppif* gene which encodes CyPD [175]. In these mice, an improved mitochondrial ATP synthesis was matched by PTP inhibition and significant suppression of motor neuron death throughout disease, although survival was not improved confirming the role of ATP synthesis and permeability transition in ALS neuron cell death [176, 177]. Increased ATP synthesis in this mouse ALS model lacking CyPD could also be explained by our observation that ablation of CyPD increases the catalysis of ATP synthase [11].

### 7.2. Alzheimer's Disease

AD, the most common form of dementia in aged people, is characterized by deposition of amyloid plaques formed by the amyloid *β* peptide [178]. Amyloid *β* can be imported in mitochondria [179]. Several studies have observed activity changes in key mitochondrial enzymes in AD. While the exact mechanism for this loss of activity is unclear, evidence suggests that ROS/RNS production is increased in AD. Nitrotyrosine residues have been detected in postmortem Alzheimer's tissue but not in age-matched controls [180], indicating the presence of ONOO^−^. The induction of iNOS in cultured rat astrocytes causes NO-mediated neuronal death in a coculture system [181].

Amyloid *β* is also reported to stimulate glial NO production [182–184] and it has been shown to inhibit both purified complex IV [185] and complex IV in isolated brain mitochondria [186]. Additionally, in neuronal cultures amyloid *β* caused a loss of activity of all the mitochondrial complexes and a loss of mitochondrial integrity, due to PTP opening [187]. It has been shown that amyloid *β* oligomers alter calcium homeostasis [188].

Upon import in mitochondria, amyloid *β* interacts with CyPD and enhances PTP induction, since neurons from CyPD KO mice are protected from cell death induced by amyloid *β* dependent PTP opening [189]. Interestingly, a novel association with AD risk has been recently identified in the ATP synthase* ATP5H* locus, which encodes subunit d of the lateral stalk [190]. Intriguingly, in a proteomic analysis of human heart mitochondria in normal condition, a tryptophan residue at the level of d subunit was identified as a “hot spot” for oxidation [36], the latter being another important pathophysiological factor in AD. Moreover, in the filamentous fungus* Podospora anserina*, a model with a clear defined mitochondrial etiology of aging, another tryptophan residue on the *α* subunit of ATP synthase (Trp503) has been described to be crucial for the selective targeting of oxidative damage [107]. Although this residue is not conserved in unicellular fungi and higher eukaryotes, the example of* P. anserina* is instructive because the authors proposed that oxidized cysteine and methionine can be efficiently reduced by repair mechanisms, whereas tryptophan oxidation products are irreversible and have the potential to form markers detected by the mitochondrial quality control system.

### 7.3. Multiple Sclerosis

In multiple sclerosis (MS), the myelin sheath of neurons in the central nervous system is destroyed leading to axonal degeneration [191]. This is associated with mitochondrial calcium overload and bioenergetic dysfunction [192]. CyPD KO mice with autoimmune encephalomyelitis display a marked protection from axonal degeneration and a milder clinical picture despite a normal inflammatory response, thus suggesting that PTP might be involved in disease pathogenesis [193], even if other effects on bioenergetics caused by CyPD ablation cannot be excluded. A further indication of the important role played by the PTP in MS and its activation by ROS is provided by the observation that axonal damage of mice undergoing experimental autoimmune encephalomyelitis is reduced by genetic ablation of p66^Shc^ [194]. Importantly, a proteomic study of experimental autoimmune encephalomyelitis identified the ATP synthase dimer-forming subunit e differentially expressed [195]. Moreover, mutations at the level of the genes encoding subunits a and A6L were observed in MS patients [196].

A large body of evidence exists implicating increased generation of RNS in MS. The observation that the concentration of NO_2_^−^ plus NO_3_^−^ in cerebrospinal fluid is elevated by 70% in MS patients supports this hypothesis [197]. Furthermore, increased iNOS activity and iNOS mRNA have been demonstrated in astrocytes associated with demyelinating lesions in postmortem MS brain [198] and in experimental models of demyelination [199, 200]. Nitrotyrosine residues indicating the presence of ONOO^−^ have also been detected in MS brain [201]. Whether NO-mediated mitochondrial damage is the cause of the disease remains to be established; however, the first direct evidence of altered mitochondrial function in MS came from a study by Lu et al. [202]. These authors described a decrease of respiratory complex I activity and a compensatory increase in complex IV in MS. Major targets for nitrosative stress in rat liver under in vivo conditions are tyrosine 345 and tyrosine 368 in the *β* subunit of the mitochondrial ATP synthase that is suggested as an early marker of nitrosative stress and aging [35]. A comparison between *α* and *β* subunits allowed the speculation that the latter is more accessible for RNS modification and that the catalytic conformation of this subunit also affects tyrosine residues exposure to nitration (higher exposure to RNS modifications in the absence of bound nucleotide). Moreover, Ding et al. [203] demonstrated in ND4 mice (an in vivo model for MS) an impaired transport of ATP5b mRNA (which generates subunit *β*) to mitochondria. This caused decreased ATP synthesis in MS mice due to lower levels of this subunit and not to impaired ATP synthase assembly, as suggested by the authors. However, the possibility that tyrosine modifications in this subunit might affect enzyme activity cannot be excluded.

### 7.4. Parkinson's Disease

PD is caused by death of dopaminergic neurons in the* substantia nigra pars compacta*. A sensitization to PTP opening has been proposed as a major cause of neurodegeneration in several models of the disease characterized by altered homeostasis of intracellular calcium [204–206], including the forms caused by complex I inhibition [207, 208] and by inactivation of the Ser/Thr kinase PINK1 [209], in which changes in calcium storage capacity [210, 211], impairment of respiratory complex I [212], and altered mitophagy [213] are observed. Moreover, the PTP can be induced in dopaminergic neurons because of the inability to buffer increased intracellular ROS levels [214]. In a PD mouse model induced by parkin deficiency, state 3 and state 4 respiration rates were both affected indicating a more likely direct modulation of the respiratory chain compared to an effect on ATP synthase [215]. On the other hand, a mouse model lacking chaperone protein* HtrA2* and showing a parkinsonian phenotype displays mitochondrial uncoupling at the level of ATP synthase and a truncated *α* subunit that might be involved in the neurodegeneration observed in these mice [33].

Postsynaptic density protein 95 (PSD-95) binds to neuronal nNOS and the neuroprotective effects of small-molecule inhibitors of this interaction were tested in an in vitro PD model. The observed protective effects were associated with suppressed mitochondrial dysfunction, as evidenced by decreased ROS generation, preserved ATP synthesis, and PTP inhibition [216]. Moreover, Darios et al. [217] demonstrated that in PC12 cells the overexpression of parkin protects from ceramide-induced swelling suggesting that parkin may act directly to prevent PTP mediated cell death, but the exact mechanism remains to be addressed.

## 8. Conclusions

Posttranslational modifications of ATP synthase due to ROS/RNS generation might play a dual role by promoting cell death or survival depending on their relative effects on mitochondrial ATP synthase catalysis and PTP. We have illustrated how ATP synthase is the target for oxidative/nitrosative modifications that affect its activity and might promote formation of the PTP. Identification of specific residues involved in the latter event is still lacking and will help to elucidate the mechanisms that mediate the role of ATP synthase in modulating cell survival or death. Finally, we discussed how changes in the ATP synthase activity regulate mitochondrial ROS formation and thus may represent an attractive strategy for the treatment of pathologies such as cancer.

## Figures and Tables

**Table 1:** Residues of ATP synthase modified by ROS/RNS in pathophysiology.

Residue	Condition	Modification	Reference(s)
*α*-Cys201	DHF	Oxidation, S-S bonds	[26]

*α*-Cys251	DHF	Oxidation, S-S bonds	[26]
	Rat brain and liver	S-glutathionylation	[27]
	Mouse heart overexpressing iNOS	S-glutathionylation	[28]
	Preconditioning and I/R	S-nitrosylation,	[29, 30]
	Aging-mouse skeletal muscles	Carbonylation,	[31]
	Rat brain mitochondria	Tyr-phosphorylation	[32]

*α* subunit	PD, *HtrA2* KO mouse	Truncation	[33]
*γ*-Cys78	DHF	Oxidation, S-S bonds	[26]

OSCP-Cys118	Bovine heart mitochondria	NEM incorporation	[34]
*β*-Tyr345	Rat liver	S-nitrosylation	[35]
*β*-Tyr368	Rat liver	S-nitrosylation	[35]
d-Trp	Human heart mitochondria	Oxidation	[36]

Residues refer to bovine ATP synthase subunits without mitochondrial targeting sequence.

DHF: dyssynchronous heart failure; iNOS: inducible nitric oxide synthase; I/R: ischemia/reperfusion; PD: Parkinson's disease; KO: knockout; OSCP: oligomycin-sensitivity conferring protein; and NEM: N-ethylmaleimide.

## Abbreviations

AD: Alzheimer's disease
ALS: Amyotrophic lateral sclerosis
AsO: Arsenite anion
BH4: Tetrahydrobiopterin
CRT: Chronic resynchronization therapy
CsA: Cyclosporin A
CuCl_2_: Copper(II) chloride
CyPD: Cyclophilin D
DHF: Dyssynchronous heart failure
eNOS: Endothelial nitric oxide synthase
GSNO: S-Nitrosoglutathione
H_2_O_2_: Hydrogen peroxide
IF_1_: Inhibitor protein
IMM: Inner mitochondrial membrane
iNOS: Inducible nitric oxide synthase
IPC: Ischemic preconditioning
I/R: Ischemia/reperfusion
KO: Knockout
MAO: Monoamine oxidase
MBM: Monobromobimane
mGSK3: Mitochondrial glycogen synthase kinase 3
PTP: Mitochondrial permeability transition pore
MS: Multiple sclerosis
NEM: N-Ethylmaleimide
nNOS: Neuronal nitric oxide synthase
NO: Nitric oxide
NO_2_^−^*⁡*: Nitrite
NO_3_^−^*⁡*: Nitrate
Nox4: NADPH oxidase 4
OMM: Outer mitochondrial membrane
ONOO^−^: Peroxynitrite
OSCP: Oligomycin-sensitivity conferring protein
PD: Parkinson's disease
PhAsO: Phenylarsine oxide
RNS: Reactive nitrogen species
ROS: Reactive oxygen species
SOD: Superoxide dismutase
SPG7: Spastic paraplegia 7.
